# Analysis of histological and microRNA profiles changes in rabbit skin development

**DOI:** 10.1038/s41598-019-57327-5

**Published:** 2020-01-16

**Authors:** Haisheng Ding, Guanglong Cheng, Jianjian Leng, Yongxin Yang, Xiaowei Zhao, Xiaofei Wang, Yunxia Qi, Dongwei Huang, Huiling Zhao

**Affiliations:** Anhui Key Laboratory of Livestock and Poultry Product Safety Engineering, Institute of Animal Husbandry and Veterinary Medicine, Anhui Academy of Agricultural Sciences, Hefei, 230031 People’s Republic of China

**Keywords:** Genetics, Genomics

## Abstract

The periodic regrowth of rabbit fur is economically important. Here, we aimed to characterise the histological traits and microRNA (miRNA) expression profiles in the skin tissue of Wan Strain Angora rabbits at different weeks after plucking. Haematoxylin-eosin staining showed that hair follicles were in the telogen phase in the first week, while they were in the anagen phase from the fourth to twenty-fourth weeks. In addition, two small RNA libraries derived from skin samples of Wan Strain Angora rabbits at telogen and anagen stages yielded over 24 million high-quality reads. Specifically, 185 miRNAs were differentially expressed between the telogen and anagen phases. The function of the differentially expressed miRNAs was explored by comparing them with known mammalian miRNAs and by Gene Ontology and Kyoto Encyclopedia of Genes and Genomes pathway analysis of their predicted targets. Five new functional miRNAs were validated using quantitative real-time PCR. Moreover, the fibroblast growth factor 5 (*FGF5*) gene was verified to be a target of conservative_NC_013672.1_9290 and conservative_NC_013675.1_10734. We investigated differential miRNA profiles between the telogen and anagen phases of the hair cycle and our findings provide a basis for future studies focusing on the mechanisms of miRNA-mediated regulation of rabbit hair follicle cycling.

## Introduction

The growth and development of hair follicles are cyclical throughout a rabbit’s life. In adults, hair follicles undergo cyclical bouts of growth (anagen), destruction (catagen), and rest (telogen), which are collectively known as the hair cycle^1–4^. Each growth period has a specific activated/silenced gene expression pattern^5^. Hence, understanding gene expression patterns can help to elucidate the regulatory mechanisms of the rabbit hair cycle.

Hair follicle development is controlled at several levels, including epigenetic regulation and transcription factor-induced signalling^6,7^. miRNAs are small (22 nucleotides) noncoding regulatory RNAs that reduce stability and/or the translation of specific mRNA targets with full or partial complementary sequences^8–10^. However, compared with other mammals, including humans (*Homo sapiens*), mice (*Mus musculus*), and rats (*Rattus norvegicus*), relatively few miRNAs in rabbits have been verified and deposited in the public miRBase database. Thus, it is necessary to clarify the roles of miRNAs in the regulation of the hair cycle in rabbits.

Emerging evidence indicates that miRNAs are involved in skin and hair follicle development. miRNA/mRNA regulatory networks are reported to be involved in the regulation of hair follicle development and epidermal homeostasis^11^. In particular, hair follicle development is regulated by miR-195-5p-induced inhibition of the Wnt/β-catenin pathway through targeting LRP6 and signature genes of Wnt signalling^12^. Furthermore, miR-339-5p negatively regulates loureirin A-induced hair follicle stem cell differentiation by targeting DLX5^13^. In addition, miR-31 is a marker of the hair growth phase, and its loss promotes hair growth^14^. Moreover, miRNAs are involved in the regulation of signalling pathways and factors related to skin development and the hair cycle.

miR-21 is an important downstream component of BMP signalling involved in skin development and tumorigenesis^15^. miR-214 regulates skin morphogenesis and hair follicle cycling by targeting β-catenin and is a key regulator of Wnt signalling and stem cell functions during normal tissue homeostasis, regeneration, and aging^16^. Thus, changes in miRNA expression patterns are closely related to hair follicle development and cycling^17^. These findings also suggest that miRNAs regulate gene expression during follicle development and hair cycling. Although substantial progress has been made in discovering important regulators of these processes in humans, pigs, mice, and sheep^8,18–21^, relatively few studies have focused on the hair cycle in rabbits^22^.

Wan Strain Angora rabbits, of Chinese origin, are used for high-yield, high-quality, and high-efficiency fur production. They were generated by crossing Germanic Angora with White New Zealand rabbits after systematic breeding^23,24^. Rabbits generate very dense hairs from primary and secondary hair follicles in the skin^25,26^. The mechanisms by which miRNAs regulate hair follicle cycling in Wan Strain Angora rabbits, however, remain largely unclear.

In the present study, we reconstructed the cycling of rabbit hair growth by hair plucking and characterized the hair cycle. In addition, small RNA-Seq analysis was conducted to investigate miRNAs profiles in skin tissues from Wan Strain Angora rabbits in the first and eighth weeks after plucking to study the function of miRNAs in the control of hair cycle. Our study allowed us to identify a large number of novel regulatory miRNAs in the hair cycle and will form a solid foundation for further exploration of the regulatory role of miRNA in the hair growth cycle.

## Results

### Characterization of the hair cycle

**Figure 1:**
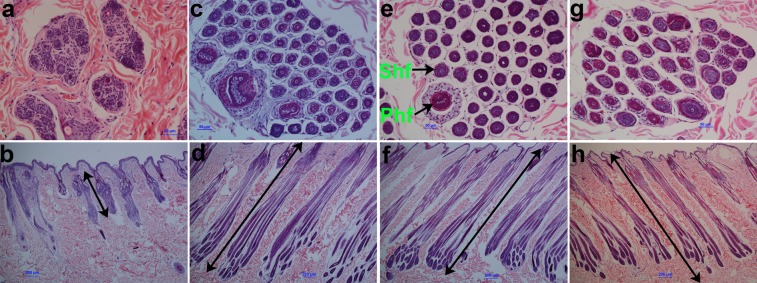
Histological observation of skin tissues from Wan Strain Angora rabbits at different weeks after hair plucking. (**a**,**b**) Transverse and longitudinal skin sections of Wan Strain Angora rabbits in the first week, respectively. (**c**,**d**) Transverse and longitudinal skin sections of Wan Strain Angora rabbits in the fourth week, respectively. (**e**,**f**) Transverse and longitudinal skin section of Wan Strain Angora rabbits in the eighth week, respectively. (**g**,**h**) Transverse and longitudinal skin sections of Wan Strain Angora rabbits in the twenty-fourth week, respectively. Phf, Primary hair follicles, Shf, Secondary hair follicles.

In order to characterize the hair cycle, we compared the follicle morphology of Wan Strain Angora rabbits at different weeks after hair plucking. A morphological analysis showed an atrophic hair follicle structure in the first week (Fig. 1a,b). In contrast, a complete hair follicle structure and an obviously increased number of hair follicles were observed in the fourth, eighth, and twenty-fourth weeks (Fig. 1c–h). Moreover, the length of hair follicles in the first week was evidently shorter than those in the fourth, eighth, and twenty-fourth weeks (Fig. 1b,d,f,h). Similar lengths of hair follicles between the fourth, eighth, and twenty-fourth weeks were observed (Fig. 1d,f,h). These results indicated that hair follicles of Wan Strain Angora rabbits were in the telogen phase in the first week after plucking, whereas they were in the anagen phase from the fourth to twenty-fourth weeks, leading to continuous hair growth throughout the period.

### Analysis of small RNA libraries

To obtain a comprehensive view of small RNA (sRNA) profiles of hair follicles in the telogen and anagen phases, two libraries were constructed from Wan Strain Angora rabbits in the first and eighth weeks after plucking, respectively, and sequenced. A total of 51,400,130 and 42,462,080 raw reads were obtained from skin tissues in the first and eighth weeks, respectively (Table 1). After removing low-quality reads, adaptors, and reads shorter than 18 nt and longer than 30 nt, 25,995,008 and 24,066,306 clean reads, ranging from 18 to 30 nt, were obtained from skin tissue in the first and eighth weeks, respectively, and used for further analysis. Then rRNA, tRNA, snRNA, snoRNA, ncRNA, and repeat sequences were filtered, and unannotated reads, including miRNAs accounting for over 61.19% of total sequences were obtained (Table 2). As a result, 14,173,689 and 13,826,004 clean reads were mapped to the rabbit genome using the Bowtie alignment software^27^ (Supplementary Table S1). Read length distributions of the two libraries were shown in Supplementary Fig. S1. In the eighth week after plucking, the majority of sRNAs were 21–23-nt long (>44%), and the 22-nt sRNAs were the most abundant (21.16% of the total reads), followed by 23-nt (12.66%) and 21-nt (10.25%) sRNAs. However, we observed a different sRNA distribution in Wan Strain Angora rabbits in the first week after plucking, in which 22-nt long sRNAs were the most abundant (24.70% of the total reads), followed by 30-nt long sRNAs (14.14%). Thus, the sRNA length distribution differed in the telogen and anagen stages of hair follicle growth in Wan Strain Angora rabbits.

**Table 1:** Small RNA sequence statistics for S01 and S02 libraries.

Samples	Raw reads	Low-quality reads	Containing ‘N’ reads	Length <18	Length > 30	Clean reads	Q30 (%)
S01	51,400,130	0	46,878	1,832,758	23,525,486	25,995,008	94.69
S02	42,462,080	0	40,917	2,813,782	15,541,075	24,066,306	95.14

S01 represents Wan Strain Angora rabbits after plucking hairs in the first week; S02 represents Wan Strain Angora rabbits after plucking hairs in the eighth week.

**Table 2:** Classification annotation of sRNAs from sRNA-Seq data.

Types	S01	S02
Total	25,995,008 (100.00%)	24,066,306 (100.00%)
rRNA	5,082,346 (19.55%)	6,949,084 (28.87%)
scRNA	0 (0.00%)	0 (0.00%)
snRNA	18 (0.00%)	23 (0.00%)
snoRNA	330,697 (1.27%)	305,604 (1.27%)
tRNA	1,753,743 (6.75%)	1,428,922 (5.94%)
Repbase	633,357 (2.44%)	656,984 (2.73%)
Unannotated	18,194,847 (69.99%)	14,725,689 (61.19%)

### miRNA profiles of skin tissues in the telogen and anagen stages of hair follicles in Wan Strain Angora rabbits

We characterised the miRNA profiles in the telogen and anagen stages of hair follicles in Wan Strain Angora rabbits and used the miRDeep2 software to align the clean reads to precursors sequences (pre-miRNAs) in the rabbit genome^28^. The analysis revealed 5 known and 546 novel miRNAs associated with the telogen stage, whereas 8 known and 558 novel miRNAs were associated with the anagen stage, respectively (Supplementary Table S2). Since the Rfam and GenBank databases contain few known rabbit miRNAs, the novel miRNAs were compared with known mammalian miRNAs (mature miRNAs) in the miRBase 21.0 database to expand the subset of “known” miRNAs in rabbit skin. As a result, 260, 265, 231, 208, 226, and 88 known miRNAs from cows, humans, mice, pigs, rats, and sheep matched the novel rabbit miRNAs from the telogen stage, respectively (Table 3), whereas 263, 264, 232, 208, 226, and 88 known miRNAs from cows, humans, mice, pigs, rats, and sheep matched the novel miRNAs from the anagen stage, respectively.

**Table 3:** The number of known mature miRNAs compared with known mammalian miRNAs.

Sample	Cow	Human	Mouse	Pig	Rat	Sheep	Total	DE miRNAs
S01	260	265	231	208	226	88	242	43
S02	263	264	232	208	226	88	242	43

The above-mentioned known and novel miRNAs were examined for differential expression, and 185 miRNAs with significantly different expression (DE miRNAs; false discovery rate (FDR) ≤0.01, |log2FC| ≥1) between the telogen and anagen stages were detected. These comprised 41 miRNAs that were highly expressed in the telogen stage and 144 miRNAs highly expressed in the anagen stage (Supplementary Fig. S2; Supplementary Data 1). In addition, 43 known mature miRNAs compared with other mammals were differentially expressed between the telogen and anagen stages, including mir-17-5p, mir-31, mir-34b, mir-34c, mir-21, and mir-20a (Table 3; Supplementary Data 2). The results demonstrated the different roles of DE miRNAs in regulating the hair cycle and revealed a large number of novel miRNAs controlling the hair cycle in Wan Strain Angora rabbits.

### Validation of differentially expressed miRNAs with quantitative real-time PCR

**Figure 2:**
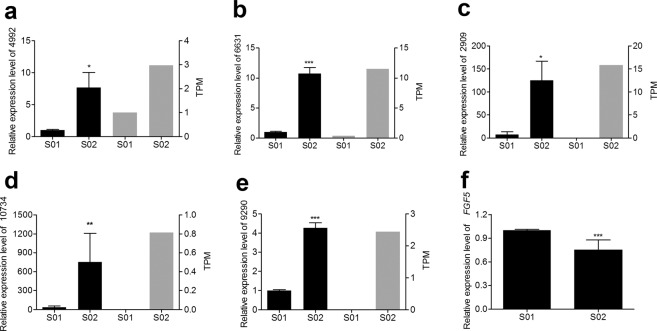
Validation of the sequencing results by q-PCR. (**a**) Conservative_NC_013686.1_4992, (**b**) Unconservative_NC_013669.1_6631, (**c**) Conservative_NC_013682.1_2909, (**d**) Conservative_NC_013675.1_10734, (**e**) Conservative_NC_013672.1_9290, (**f**) FGF5. In panels (**a**–**e**), the black and grey columns represent the q-PCR and sequencing results, respectively. S01 represents Wan Strain Angora rabbits after plucking hairs in the first week; S02 represents Wan Strain Angora rabbits after plucking hairs in the eighth week. TPM, transcript per million. *P < 0.05; **P < 0.01; ***P < 0.001.

To validate the sRNA-Seq results, q-PCR was performed to investigate the relative expression levels of five selected miRNAs (conservative_NC_013686.1_4992, unconservative_NC_013669.1_6631, conservative_NC_013682.1_2909, conservative_NC_013675.1_10734, and conservative_NC_013672.1_9290). As shown in the histogram (Fig. 2a–e), the data pertaining to the sRNA-Seq and q-PCR analyses were nearly identical. In addition, the relative expression level of the predicted target gene for conservative_NC_013672.1_9290 and conservative_NC_013675.1_10734, *FGF5*, was evaluated in the skin tissue during the telogen and anagen stages (Fig. 2f). The analysis revealed that *FGF5* expression was inversely correlated with that of the two miRNAs.

### miRNA target gene prediction, verification, and functional annotation analyses

**Figure 3:**
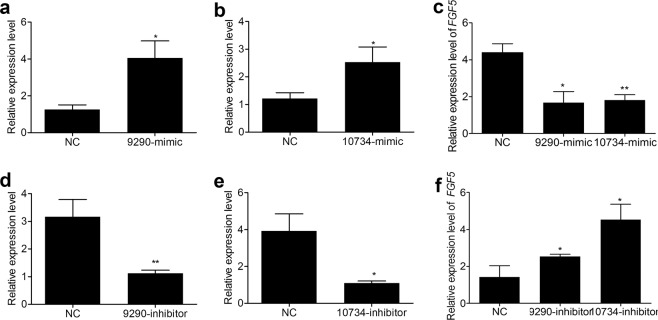
Identification of *FGF5* as a target of conservative_NC_013672.1_9290 and conservative_NC_013675.1_10734 in RAB-9 cells. (**a**,**b**) Relative expression of conservative_NC_013672.1_9290 and conservative_NC_013675.1_10734 after transfecting mimics, respectively. (**c**) Relative expression of endogenous *FGF5* mRNA after transfecting conservative_NC_013672.1_9290 and conservative_NC_013675.1_10734 mimics. (**d**,**e**) Relative expression of conservative_NC_013672.1_9290 and conservative_NC_013675.1_10734 after transfecting inhibitors, respectively. (**f**) Relative expression of *FGF5* mRNA after transfecting conservative_NC_013672.1_9290 and conservative_NC_013675.1_10734 inhibitors.

In order to gain insights into the roles of the identified miRNAs in the hair cycle, the potential targets of the DE miRNAs were predicted. Specifically, 6046 and 20554 target genes were predicted for the 10 known and the 562 novel miRNAs, respectively (Supplementary Table S3). *FGF5* was predicted as the common target gene of the two DE miRNAs, conservative_NC_013672.1_9290 and conservative_NC_013675.1_10734. q-PCR analyses revealed that *FGF5* mRNA expression was significantly suppressed after transfecting conservative_NC_013672.1_9290 and conservative_NC_013675.1_10734 mimics into RAB-9 cells (Fig. 3a–c). Consistently, inhibition of these two miRNAs increased *FGF5* mRNA (Fig. 3d–f), indicating that *FGF5* gene was a target of the two miRNAs.

**Figure 4:**
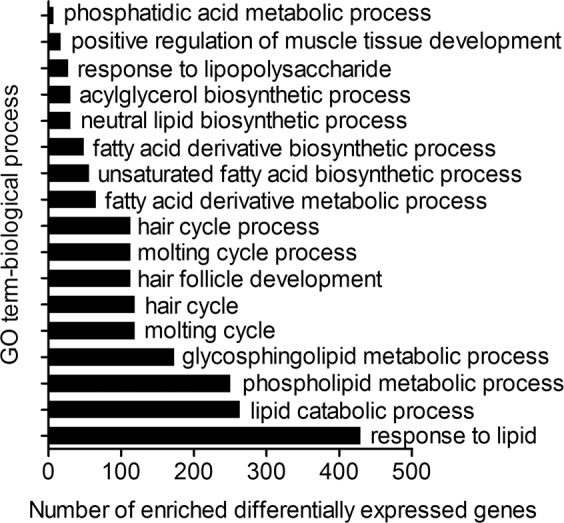
Significantly enriched GO terms for target genes of DE miRNAs between telogen and anagen stages (*P*<0.05). The hierarchical category of the GO terms is “biological process”. The x-axis represents the number of target genes enriched in the biological processes; the y-axis represents the GO terms enriched by target genes. DE miRNAs, differentially expressed miRNAs.

In addition, Gene ontology (GO) and pathway enrichment analyses were used to explore the function of DE miRNAs in the regulation of hair follicle cycling. As for the biological process category, GO term annotation results showed that hair follicle development, hair cycle, and lipid catabolism were significantly enriched by the targets of DE miRNAs (Fig. 4), suggesting that these miRNAs may be involved in regulating hair follicle development and lipid metabolism. Kyoto Encyclopedia of Genes and Genomes (KEGG) analysis showed that TGF-β signalling, Wnt signalling, ECM-receptor interactions, apoptosis, as well as fat digestion and absorption pathways, were enriched by targets of DE miRNAs (Supplementary Table S4), suggesting the potential involvement of the relevant miRNAs in the regulation of hair follicle development and cycling in Wan Strain Angora rabbits.

## Discussion

Hair growth occurs periodically in a cycle that consists of three different phases: growth (anagen), regression (catagen), and resting (telogen)^25,29^. These phases may be distinguished according to hair follicle morphology. In our previous study, we reconstructed the follicle cycle of rabbit hair growth by plucking hairs and measured the hair length of Wan Strain Angora rabbits at different weeks after plucking^30^. The results showed that rabbit hairs grew in a linear growth rhythm from the fourth to twenty-fourth weeks for different types of hair, suggesting that hair follicles were still in the anagen phase until the twenty-fourth week after plucking.

In the present study, we identified changes in follicle morphology of Wan Strain Angora rabbits after plucking. Hair follicles entered the short telogen phase in the first week and were in anagen phase from the fourth to twenty-fourth weeks, which was consistent with our previous study on hair growth^30^. During the telogen phase, hair follicle stem cells are relatively quiescent, and the bulge of the hair follicles is in contact with the dermal papilla^29^. In the anagen phase, hair follicle stem cells are activated and rapidly proliferate^3,31^. Hair growth is fuelled by stem cells activated at the beginning of the anagen phase by the dermal papilla^3^. In this study, the length of hair follicles in the first week was found to be evidently lower than those in the fourth, eighth, and twenty-fourth weeks. At these later stages, hair follicle length was similar, indicating that hair follicles were activated between the first and fourth weeks and were maintained in the anagen phase until the twenty-fourth week. The long anagen phase of the hair cycle promoted the continuous growth of Wan Strain Angora rabbit hair.

miRNA–mediated regulation is a critical and effective mechanism underlying the development of skin and hair follicles. The transitions between phases in the dynamic hair cycle are influenced by interactions between a series of growth signals and inhibitory molecules controlling the growth of hair follicles. Recently, the intragenic mRNA-miRNA regulatory network during the telogen–anagen transition was investigated, demonstrating that hair follicle growth initiation and development were related to miR-195 and the genes *CHP1*, *SMAD2*, *FZD6*, and *SIAH1* in the cashmere goat^5^. Here, to elucidate the molecular mechanisms regulating hair follicle cycling, miRNA expression profiles were investigated in the skin tissue of Wan Strain Angora rabbits, after reconstructing hair follicle cycling.

Over 24 million clean reads were derived, which is consistent with recently reported results^22^. The read length distributions of two small RNA libraries, corresponding to distinct stages of hair follicle growth, demonstrated that 22-nt long sequences were the most represented, which was in accordance with the normal size of miRNAs reported in a previous study^32^. In addition, 30-nt reads may represent Piwi-interacting RNAs (piRNAs)^33,34^. Many piRNAs were detected in skin tissues of Wan Strain Angora rabbits and, notably, clearly decreased in the eighth compared with the first week after plucking, suggesting their involvement in the telogen–anagen hair follicle transition. However, the mechanisms by which piRNAs regulate the hair cycle need further investigation.

The miRNA expression profiles were compared between the telogen and anagen stages, and 185 DE miRNAs were detected. This set did not include known rabbit miRNAs. After comparing with known mammalian miRNAs, 43 DE rabbit miRNAs were found to be conserved among various species. Thus, the remaining 142 DE miRNAs were considered to be novel functional miRNAs potentially regulating the hair cycle. The regulatory roles of the new DE miRNAs may be inferred from their target genes and relative expression patterns. Consequently, we carried out a prediction of target genes and verified that *FGF5* was a target gene of conservative_NC_013672.1_9290 and conservative_NC_013675.1_10734 miRNAs. *FGF5* serves as a crucial regulator in hair length^35,36^ and influences the hair cycle by regulating the anagen–catagen transition^35,37–39^. Our results indicated that conservative_NC_013672.1_9290 and conservative_NC_013675.1_10734 were candidate regulatory miRNAs in the hair cycle.

GO analysis showed that a large proportion of target genes of the DE miRNAs were significantly enriched in the biological process category, including the hair cycle, hair follicle development, and lipid catabolism. Thus, the DE miRNAs were potential regulators of hair follicle development and lipid metabolism in rabbits. In addition, miRNAs may regulate the hair cycle through key genes controlling signalling pathways that modulate hair follicle growth^5,40^. The MAPK signalling pathway, Wnt signalling pathway, ECM-receptor interaction, as well as fat and fatty acid metabolism pathways, were enriched by target genes of DE miRNAs. The MAPK signalling pathway is involved in the regulation of catagen entry and quiescence of hair follicle stem cells^39^. Wnt signalling is a key regulator of hair follicle morphogenesis and life-long hair follicle regeneration^41^, and regulates the initiation of hair follicle development^42^. Wnt proteins are pivotal in conveying inductive signals between the follicular mesenchyme and epithelium^43^. ECM-receptor interactions are essential for the morphogenesis of hair follicles and potentially regulate follicle development and hair growth^44,45^.

The results of our study indicated that the morphology of hair follicles changed during the telogen–anagen transition. ECM-receptor interactions may be involved in controlling hair follicle structural homeostasis and cycling in the skin of Wan Strain Angora rabbits. Our observation that a large proportion of target genes of newly identified DE miRNAs were implicated in fatty acid metabolism was in line with previous studies reporting that lipid metabolism is a major determinant of wool diameter and hair growth^44,46^, consistent with a role of fat and fatty acid metabolism in the hair cycle.

However, there are some important limitations of the present study that should be highlighted. Firstly, considering animal welfare, we only conducted histological analysis of Wan Strain Angora rabbits in the first, fourth, eighth, and twenty-fourth weeks after plucking hairs, and the possible histological changes in the period from the eighth to twenty-fourth weeks were ignored. It helps us to better understand the histological morphology of hair follicles and the growth rhythm of rabbit hair at each stage if more hair follicle development stages were studied. Our previous study reconstructed the hair cycle by plucking hairs and showed that hair growth continued from the eighth to twenty-fourth week by measuring hair length once every two weeks^30^. In addition, because there were no suitable antibodies, we did not quantify *FGF5* protein content to augment the results. In future research, we aim to prepare monoclonal antibodies of rabbit *FGF5* for protein content quantification. The regulatory mechanism underlying conservative_NC_013672.1_9290 and conservative_NC_013675.1_10734 in the hair cycle remains unclear in rabbits. Next, we will perform functional analyses of the two miRNAs at the cellular level and *in vivo*.

In summary, this study has identified differences in hair follicle histology and miRNA profiles between the telogen and anagen phases of the hair cycle. Further, new miRNAs, potentially involved in the rabbit hair cycle, were revealed. This study serves as the basis for functional studies addressing the role of miRNAs in the rabbit hair cycle.

## Materials and Methods

### Animals

The experiments were performed in 1.5-year-old Wan Strain Angora rabbits, about 4.0 kg in average weight. The rabbits were procured from the rabbit farm of the Institute of Animal Husbandry and Veterinary Medicine of Anhui Academy of Agriculture Sciences, Hefei, Anhui, China, raised and managed under the same conditions, feeding pellets restrictedly and drinking water *ad libitum*. This study was carried out in strict accordance with relevant guidelines and regulations by the Ministry of Agriculture of the People’s Republic of China. All experimental protocols were approved by the Ethics Committee of Anhui Academy of Agricultural Sciences.

### Sample collection, preparation, and histological examination

Ten hours before plucking, three rabbits were treated with oral dexamethasone 1.5 mg and about 30 cm^2^ hair was plucked to initiate a new hair cycle. Oral dexamethasone dilated hair follicles and made hair easily to be pulled out. Meanwhile, the manipulation should be done correctly and the strength should be moderate when pluck hairs. Hold the rabbit in place with your left hand, pinch the hair fibers with your index, middle and thumb fingers of right hand, and pull it out a pinch by a pinch. Rabbits were given anaesthesia through an ear vein injection of 0.7% pentobarbital sodium (6 ml/kg) before sampling. Skin tissue samples (about 1 cm^2^) from the back of each rabbit were collected in the first, fourth, eighth, and twenty-fourth weeks after plucking hairs. The skin samples were fixed in 4% paraformaldehyde solution for histological analysis. Cross sections of the fixed samples were washed with running water, dehydrated using an ethyl alcohol series, cleaned in xylene, and embedded in paraffin wax. The specimens were sectioned to a thickness of 4 μm using a Leica RM2235 microtome (Leica, Wetzlar, Germany). Transverse and vertical cross-sections of the fixed and paraffin-embedded samples were stained with haematoxylin-eosin (HE), examined, and photographed using an Olympus BX51 biomicroscope (Olympus Optical Company, Tokyo, Japan).

### Small RNA library construction, sequencing and analysis

The whole skin including the dermis and dermal cells was used for sequencing^5,40,43^, and hair follicles were the parts of the dermis. Skin samples were isolated from the dorsal skin of three Wan Strain Angora rabbits in the first week after hair plucking (S01 samples), when the wool cycle is expected to be in the telogen phase, and in the eighth week post-plucking (S02 samples), i.e., in the anagen phase. Samples were frozen in liquid nitrogen immediately and stored at −80 °C prior to RNA extraction.

Total RNA was extracted from S01 and S02 skin samples using TRIzol reagent (Invitrogen, Carlsbad, CA, USA), according to the manufacturer’s instructions. The samples were sent to the Beijing Biomarker Technologies Company. Two small RNA (sRNA) libraries were constructed and generated using the Next Ultra small RNA Sample Library Prep Kit from Illumina (Illumina, San Diego, CA, USA), following the manufacturer’s recommendations. The sRNA fragments (18–30-nt), isolated from RNA pools by polyacrylamide gel electrophoresis (PAGE) and ligated with 5′ and 3′ adaptors, were reverse-transcribed and amplified. The libraries were quantified using an Agilent 2100 Bioanalyzer (Agilent Technologies, Santa Clara, CA, USA). Each sRNA library was sequenced using an Illumina HiSeq. 2500 platform (Biomarker, Beijing, China).

The raw reads were filtered to remove low-quality reads, adaptors, and reads shorter than 18-nt or longer than 30-nt to obtain clean reads. Clean reads were mapped to Silva, GtRNAdb, Rfam, and Repbase databases to remove rRNA, tRNA, snRNA, snoRNA, and ncRNA, as well as repeat sequences, and to obtain the unannotated reads including miRNAs. The remaining high-quality sequences were subsequently aligned to rabbit miRNAs. The reads that did not match known miRNAs from rabbits were further analysed to identify novel miRNAs. Then, the novel mature miRNAs were blasted to miRNAs of other mammals (humans, mice, pigs, cows, rats, and sheep) using miRBase 21.0. miRNAs in the libraries with identical or related sequences (0 nucleotide substitutions and vacancy permitted) to other mammals were identified as known mature miRNAs.

The miRNA expression levels were estimated by TPM (transcript per million) values^47^. Differential expression analysis between groups was performed using the IDEG6 package^48^. The Audic and Claverie test was used to identify differentially expressed miRNAs. |log2 (Fold Change)| ≥1 and FDR ≤0.01 were set as the default threshold for significantly differential expression. To predict the genes targeted by miRNAs, two computational target prediction algorithms (RNAhybrid^49^ and miRanda^50^) were used to identify miRNA binding sites. GO^51^ and KEGG^52^ enrichment analyses were performed on miRNA targets.

### Cell culture and transfection analyses

RAB-9 (ATCC® CRL-1414TM) cells were cultured in Dulbecco’s modified Eagle medium (Hyclone, Logan, UT, USA) supplemented with 10% foetal bovine serum (FBS) (CLARK, Worcester, MA, USA) under 5% CO_2_ at 37 °C. The cells were plated into 6-well plates. Transfection was performed when cells were 70–80% confluent. miRNAs mimics or inhibitors (Supplementary Table S5) were transfected into the cells using lipofectamine 2000 (Invitrogen, Carlsbad, CA, USA). The cells were harvested at 48 h post-transfection.

### Quantitative real-time PCR

To verify the sRNA-Seq results, a set of five miRNAs was selected from the list of DE miRNAs and the relative expression level of each miRNA was evaluated by quantitative real-time PCR (q-PCR). The primers for q-PCR were listed in Supplementary Table S6. Total RNA was isolated by TRIzol reagent (Invitrogen, Carlsbad, CA, USA) following the manufacturer’s instructions. Reverse transcription of miRNAs and mRNAs was performed using the RevertAid First Strand cDNA Synthesis Kit (Thermo, Wuhan, k1622) in accordance with the manufacturer’s instructions. Quantitative real-time PCR was performed using 2 × SG Green qPCR Mix (with ROX), U6 and *GAPDH* were used as the internal control. Thermal cycling conditions were as follows: 10 min at 95 °C, 40 cycles of 10 s at 95 °C and 15 s at 60 °C, and a melt curve stage of 95 °C for 15 s, 60 °C for 30 s, and 95 °C for 15 s. Data were analysed by the 2^−ΔΔCT^ method. The q-PCR analysis was performed in triplicates for each sample.

### Statistical analyses

The data were presented as mean ± standard deviation (SD). All statistical analyses were performed using GraphPad Prism 5.0 software (GraphPad Software, Inc., La Jolla, CA, USA). Student’s *t*-test was used for statistical comparisons. The results with a *P* value < 0.05 were considered as indicative of statistically significant differences.

## Supplementary information

Dataset 1.

Dataset 2.

Supplementary information.

## Data Availability

The data were presented in the manuscript and the supporting materials. The raw reads data were submitted to the Short Read Archive (SRA) under the accession number SRP191476 and BioProject accession number PRJNA531523 (https://www.ncbi.nlm.nih.gov/sra/ PRJNA531523).
