# Supplementary material for: Analysis of histological and microRNA profiles changes in rabbit skin development
Source: Sci Rep. 2020 Jan 16;10:454. doi: 10.1038/s41598-019-57327-5 (PMC6965608; doi:10.1038/s41598-019-57327-5)
Supplement: Supplementary file 3 — Supplementary information. [file 41598_2019_57327_MOESM3_ESM.pdf]

Analysis of histological and microRNA profiles changes in rabbit skin development

Haisheng Ding[1], Guanglong Cheng[1], JianJian Leng[1], Yongxin Yang[1], Xiaowei Zhao[1], Xiaofei Wang[1], Yunxia Qi[1], Dongwei Huang[1*], Huiling Zhao[1*]

[1] Anhui Key Laboratory of Livestock and Poultry Product Safety Engineering, Institute of Animal Husbandry and Veterinary Medicine, Anhui Academy of Agricultural Sciences, Hefei, 230031, Anhui, People's Republic of China.

* Correspondence and requests for materials should be addressed to D.H. (email: hdwscience@163.com) or H.Z. (email: zhl11988@163.com).

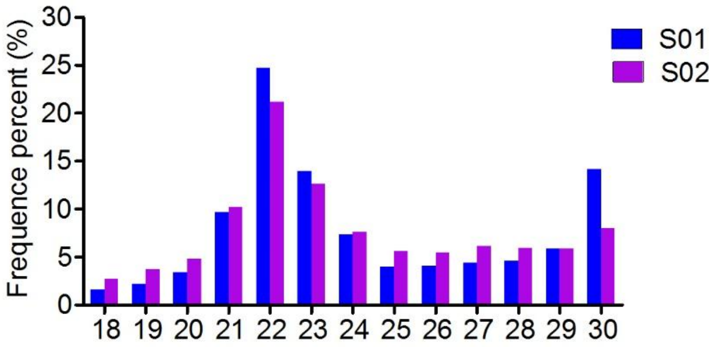

Supplementary Figure S1 Read distribution analyses of sRNAs in the skin tissue of Wan strain Angora rabbits. S01 represents Wan strain Angora rabbits after plucking hairs at the first week when the hair follicles were in the telogen stage; S02 represents Wan strain Angora rabbits after plucking hairs at the eighth week when the hair follicles were in the anagen stage.

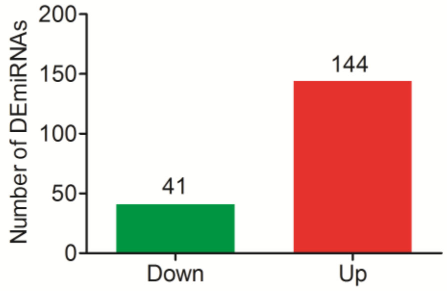

Supplementary Figure S2 Analyses of DE miRNAs between the telogen and anagen stages.

Supplementary Table S1 The number of reads mapping to rabbit reference genome from each sample. S01 represents the telogen stage; S02 represents the anagen stage.

Samples	Total Reads	Mapped Reads	Mapped reads (+)	Mapped reads (-)
S01	25,995,008	14,173,689 (54.5%)	9,544,778	4,628,911
S02	24,066,306	13,826,004 (57.4%)	10,078,636	3,747,368

Supplementary Table S2 The number of miRNAs in telogen and anagen stage, respectively. Known miRNAs, miRNAs matchped to rabbit miRBase; Novel miRNAs, miRNAs unmapped to rabbit miRBase.

Samples	Known miRNAs	Novel miRNAs	Total
S01	5	546	551
S02	8	558	566
Total	10	564	574

Supplementary Table S3 The number of targets of the miRNAs.

Types	All miRNAs	miRNA with targets	Target genes
Known miRNAs	10	10	6046
Novel miRNAs	564	562	20554
Total	574	572	20554

Supplementary Table S4 The pathways regulated hair follicle development in Wan strain Angora rabbits.

Pathways	Number of targets of DE miRNAs
TGF-β signalling pathway	84
MAPK signalling pathway	8
Wnt signalling pathway	123
ECM-receptor interaction	94
Apoptosis	59
Fat digestion and absorption	29
Fatty acid metabolism	49
Notch signalling pathway	35
Hedgehog signalling pathway	52

Supplementary Table S5 Synthesis of conservative_NC_013672.1_9290 and conservatative_NC_013675.1_10734 sequences. NC, negative control.

Sequence name	Sequence (5 ´-3 ´)
conservative_NC_013672.1_9290 mimic	GCGGCGGCGGCGACUCCG<br>GAGUCGCCGCCGCCGCUU
conservative_NC_013672.1_9290 inhibitor	GCGGCGGCGGCGACUCCG
conservatative_NC_013675.1_10734 mimic	CGGCGGCGGCCGGGCGCA<br>CGCCCGGCCGCCGCCGUU
conservatative_NC_013675.1_10734 inhibitor	CGGCGGCGGCCGGGCGCA
Negative control (NC)	UUCUCCGAACGUGUCACGUTT<br>ACGUGACACGUUCGGAGAATT
Inhibitor NC	CAGUACUUUUGUGUAGUACAA

Supplementary Table S6 Primers for qPCR. F[1], forward primer; R[2], reverse primer.

Primers	Sequences (5 ´-3 ´)
conservative_NC_013686.1_4992-F[1]	TATATTATAGGGGCGGGCGGGGCCGGC
unconservative_NC_013669.1_6631-F	TTAATACCTCCCGGGGCGGGGTGGGGG
conservative_NC_013682.1_2909-F	ATCGCAGGGGAAGTGACTTCTA
conservative_NC_013675.1_10734-F	ATTAATATTACGGCGGACGCCGGGCGCA
conservative_NC_013672.1_9290-F	ATATATAGCGGCGGCGGCGACTCCG
miRNA-R[2]	TAACGAGACGACGACAGAC
U6-F	CTCGCTTCGGCAGCACA
U6-R	AACGCTTCACGAATTTGCGT
FGF5-F	TGTGGCCTTGAACAAACGAG
FGF5-R	CTGCTCCGACTGCTTGAATC
GAPDH-F	GCCCTCAATGACCACTTTGT
GAPDH-R	TTACTCCTTGGAGGCCATGT
